# *Stagnihabitans* *lacustris* sp. nov., an Anoxygenic Photoheterotrophic Bacterium of the Family *Paracoccaceae*, Isolated from a Eutrophic Pond in Czechia

**DOI:** 10.3390/microorganisms14051157

**Published:** 2026-05-20

**Authors:** Aditi Singh, Sumeeta Kumari, Gunjan Vasudeva, Mohit Kumar Saini, Anil Kumar Pinnaka, Karel Kopejtka, Michal Koblížek, Nupur

**Affiliations:** 1 Department of Life Science, Sharda School of Bio-Science and Technology, Sharda University, Greater Noida 201306, India; 2023372186.aditi@dr.sharda.ac.in; 2 Microbial Type Culture Collection and Gene Bank (MTCC), CSIR Institute of Microbial Technology (CSIR-IMTECH), Chandigarh 160036, India; sumeetakumari20@gmail.com (S.K.); gunjanvasudeva22@gmail.com (G.V.); apinnaka@imtech.res.in (A.K.P.); 3 Laboratory of Anoxygenic Phototrophs, Centre Algatech, Institute of Microbiology CAS, 37901 Třeboň, Czech Republic; saini@alga.cz (M.K.S.); kopejk00@alga.cz (K.K.)

**Keywords:** bacteriochlorophyll *a*, *Paracoccaceae*, *Stagnihabitans*, *Rhodobacter*, anoxygenic phototrophic bacteria

## Abstract

A novel photoheterotrophic, beige-pigmented, bacteriochlorophyll *a*-containing strain KR11^T^ was isolated from Kaprový pond in Třeboň, Czechia. KR11^T^ cells were Gram-negative, rod-shaped, and motile. The isolated strain grew under photoheterotrophic conditions between 20 and 40 °C (optimum 22–25 °C), at pH ranges from 6.0 to 9.0 (optimum 7.0). It did not require NaCl for growth but tolerated NaCl concentrations up to 1.5% (*w*/*v*). No growth was observed under photoautotrophic conditions. Strain KR11^T^ showed the highest 16S rRNA gene sequence similarity to the type strains of *Stagnihabitans tardus* CYK-10^T^ (98.84%), *Tabrizicola fusiformis* SY72^T^ (95.95%), and *Rhodobacter sediminis* N1^T^ (95.37%). Phylogenetic analyses based on 16S rRNA gene sequences indicated that strain KR11^T^ clusters within the genus *Stagnihabitans* in the family *Paracoccaceae* of class *Alphaproteobacteria.* The whole-genome sequence of strain KR11^T^ comprises 4,085,976 bp with a 65 mol% G+C content. Phylogenomic analysis, including core-genome phylogeny, and the low genomic similarity (<95% ANI and <70% dDDH) to phylogenetically related taxa confirmed the taxonomic separation of strain KR11^T^ at the species level. The distinctive phenotypic traits, chemotaxonomic studies, phylogenetic, and genomic analysis establish strain KR11^T^ as a novel species within the genus *Stagnihabitans.* Accordingly, we propose the name *Stagnihabitans lacustris* sp. nov. KR11^T^ (=CCUG 74777^T^, LMG 31924^T^), isolated from fresh water.

## 1. Introduction

The *Rhodobacteraceae* family, previously defined by Garrity et al., 2005, belongs to the order *Rhodobacterales*, class *Alphaproteobacteria*, and includes bacteria with different phenotypic, metabolic, and ecological properties [1]. The *Rhodobacteraceae* family contains many phototrophic species that harvest light using bacteriochlorophyll (BChl) *a* and carotenoids of the spheroidene series. In 2021, Liang et al. separated the marine *Roseobacter* clade into the new family *Roseobacteraceae*, based on whole-genome phylogenetic analysis from family *Rhodobacteraceae* [2]. The remaining taxa in *Rhodobacteraceae* was found to bear an illegitimate name, contravening Rule 51 of the International Code of Nomenclature of Prokaryotes, and was formally replaced by the name *Paracoccaceae*, with *Paracoccus* as the type genus [2] and validated by Göker 2022 [3]. Huang et al. subsequently performed a comprehensive genome-based reclassification of genera within the family *Paracoccaceae* using phylogenomic analysis of 1606 high-quality genomes and AAI metrics, identifying cases of misclassification arising from over-reliance on 16S rRNA gene phylogeny alone [4]. Strain KR11^T^ belongs to the genus *Stagnihabitans* within the family *Paracoccaceae*, class *Alphaproteobacteria* and order *Rhodobacterales*.

The genus *Stagnihabitans* (earlier classified in *Rhodobacter* genus) was proposed by Ma et al. 2022, based on analyses of 16S rRNA, *gyrB*, and concatenated protein phylogenetic trees together with genome comparisons [5]. To date, only one species has been validated in the genus *Stagnihabitans*, named as *Stagnihabitans tardus* (http://www.bacterio.net/). *S. tardus* CYK-10^T^ was isolated from a freshwater pond in Hinoki Village in Chiayi County, Taiwan. It is a Gram-negative, aerobic, non-motile, and ovoid to rod-shaped with ubiquinone-10 (Q-10) as the main respiratory quinone [6]. The major cellular fatty acid is C_18:1_ ω7c, and the major hydroxyl fatty acids are C_10:03_-OH and C_18:03_-OH. The DNA G+C content is 66%.

In the present study, we report the phenotypic and genomic characteristics of strain KR11^T^, which was isolated from the eutrophic Kaprový pond in Třeboň, Czechia. Chemotaxonomic and genetic analyses indicate that KR11^T^ belongs to the genus *Stagnihabitans*. Furthermore, our phylogenomic research confirmed that strain KR11^T^ comprises a new lineage within the genus *Stagnihabitans* and is clearly differentiated from its nearest relatives, hence validating its classification as a novel species and proposing the name *Stagnihabitans lacustris*.

## 2. Materials and Methods

### 2.1. Sample Collection and Isolation

In this study, a bacterial strain KR11^T^ was isolated from water samples (temperature 15 °C, pH 7.3) collected from a eutrophic Kaprový pond (48°59.47′ N, 14°46.86′ E), Třeboň, Czechia, on 29 October 2018. Water samples were serially diluted to concentrations of 10^−3^ and 10^−4^ (*v*/*v*). Further, 100 µL of each dilution was spread on R2A agar plates and incubated under microaerophilic conditions (10% O_2_ + 90% N_2_) at 28 ± 1 °C under 12 h light:12 h dark cycles. The modified fluorescence imaging system, FluorCam 800 MF (Photon Systems Instruments Ltd., Drásov, Czech Republic), was used to identify the BChl autofluorescence positive colonies on the agar plates. The BChl positive colonies were picked and transferred to fresh media. Purified strain KR11^T^ was regularly maintained on slants of R2A agar and preserved in glycerol (20% *w*/*v*) at −80 °C.

### 2.2. Microscopic and Spectroscopic Examination

Phase contrast microscopy was performed using the Axio Imager. Z2 (Carl Zeiss AG, Jena, Germany) equipped with the Zeiss Plan-NEOFLUAR 100×/1.3 oil Ph3 phase contrast objective (Carl Zeiss AG, Jena, Germany). The in vivo absorption spectra of strain KR11^T^ were recorded using the UV-2600 spectrophotometer (Shimadzu, Kyoto, Japan) equipped with an integrating sphere. To further reduce scattering, the cells were suspended in 50% (*w*/*v*) glycerol. For the in vitro absorption spectra, the pigments were extracted with acetone–methanol (7:2), filtered through a 0.2 µm polytetrafluoroethylene 4 mm filter, a single-use filter device (Whatman PLC, Marlborogh, MA, USA).

### 2.3. Physiological and Biochemical Characterization

Physiological and biochemical characterization was carried out in R2A media aerobically at 25 °C ± 1 °C under 12 h light:12 h dark cycles unless stated otherwise. Anaerobic growth was assessed using R2A agar plates kept in anaerobic jars (Anoxomat), and for microaerophilic growth, plates were kept in an incubator maintaining microaerophilic conditions (10% O_2_ + 90% N_2_). To determine the growth at different temperatures, KR11^T^ strain was inoculated on R2A agar medium at 5–50 °C (interval of 5 °C). The range of pH for growth was explored in R2A broth, which was prepared using buffer systems between 4.0 and 12.0 pH (1.0 pH unit interval) [7]. The tolerance to salt was determined by supplementing the R2A agar medium with 10% (*w*/*v*) NaCl (in increments of 1% *w*/*v*). Gram-staining of the strain KR11^T^ was accomplished by the standard Gram’s reaction [8]. Oxidase activity was determined using the filter paper method as described by Kovács, 1956 [9], whereas catalase activity was tested by observing bubble formation on the addition of 3% (*v*/*v*) H_2_O_2_ drops. Carbon source utilization was carried out at 25 °C using the minimal media containing K_2_HPO_4_ (0.5 g L^−1^), peptone (0.15 g L^−1^), 1 mL trace element solution SL-8 supplemented with one of the following carbon sources (5 mM): sucrose, inulin, sodium pyruvate, malate, glucose, glycerol, acetate, melibiose, galactose, fructose, mannose, cellobiose, trehalose, sorbitol, rhamnose, or lactose [10].

### 2.4. Chemotaxonomic Analyses

For fatty acid and polar lipid analysis, 1 g of cell biomass was obtained from a culture grown on R2A agar. The polar lipids of strain KR11^T^ were extracted and then identified through second-dimensional thin-layer chromatography [11]. The first-dimension separation was done with a mixture of chloroform/methanol/water in the ratio of (60:30:3.8, *v*/*v*), and the second-dimension with a mixture of chloroform/methanol/acetic acid/water (40:7.5:6:1.8, *v*/*v*). Polar lipids on plates were visualized using 10% ethanolic phosphomolybdic acid for total lipid analysis, alpha-naphthol with ninhydrin agent for amino lipids, and molybdenum blue for phospholipids (PLs). For fatty acids, whole cells of strain KR11^T^ were saponified, methylated, extracted, and identified using the MIDI/Hewlett-Packard Microbial Identification System (Midi, Inc., Newark, DE, USA) using the MIS library TSBA6 database, following the manufacturer’s instructions.

Respiratory quinones were identified in the same acetone/methanol extract as pigments. In total, 50 µL of the extract was injected into the Prominence-i HPLC system (Shimadzu, Kyoto, Japan). The quinones were separated on a heated (40 °C) Kinetex 2.6 µm C18 100 Å 150 × 4.6 mm column (Phenomenex, Torrance, CA, USA) using a binary solvent system, 1′ 100%A, 12′ 100%B, 24′ 100%B, 25′ 100%A, where A: 100% methanol and B: methanol + heptane 10:3 (*v*/*v*) with a flow rate of 1 mL min^−1^. The eluted quinones were detected at 270 nm and identified based on their absorption spectra recorded by a UV-VIS diode-array detector (Shimadzu, Kyoto, Japan) and the retention time determined using the Q-10 standard.

### 2.5. Genome Sequencing and Annotation

Genomic DNA was isolated from the strain KR11^T^ using ZR bacterial/fungal Miniprep kit (Zymo Research, Irvine, CA, USA) according to the instructions of the manufacturer. The identity of the culture was verified by amplification and sequencing of its 16S rRNA gene. The Illumina whole-genome shotgun sequencing was performed using Illumina HiSeq platform at MedGenome (https://diagnostics.medgenome.com/, accessed on 4 August 2020) with 2 × 150 bp chemistry at 100× sequencing depth. The raw reads were checked for quality using FastQC v0.11.9 [12]. The reads with a Phred score higher than 33 were selected and further processed. The Illumina sequences and adapters from raw reads were removed using Trimmomatic v0.39 [13]. The reads were further assembled on Spades v3.13.0 [14], and the assembled draft genome was visualized on Quality Assessment Tool (QUAST) [15]. The contamination and completeness of the draft genome was checked using CheckM v1.2.3 [16]. An annotation was carried out using NCBI Prokaryotic Genome Annotation Pipeline (PGAP v6.9) [17].

### 2.6. Phylogeny and Genomic Analyses

The phylogenetic analysis of the 16S rRNA gene was done by comparing the sequence with all validly named species using EZbioCloud [18]. The 16S rRNA gene sequence (GenBank accession number MT586031) of strain KR11^T^ was retrieved from its genome sequence (GenBank accession number JAPDGS000000000). Reference sequences were obtained from NCBI GenBank in February 2026 and aligned using MAFFT [19]. The neighbour-joining (NJ) and maximum likelihood (ML) algorithms were used to construct a phylogenetic tree in MEGA version 12 software [20]. Tree topology was evaluated with bootstrap analysis using 1000 replicates. *Deinococcus xianganensis* Y35^T^ was used as an outgroup.

The ANI of strain KR11^T^ with other reference strains was calculated using the OrthoANI toolv0.7.0 [21] and a heatmap based on ANI was generated with OrthoFinder v3.1.4 [22]. The dDDH values and differences in G+C content were calculated on the online platform Genome-to-Genome Distance Calculator (GGDC 2.1). The GGDC uses Genome BLAST Distance Phylogeny (GBDP) with advanced statistical models to generate dDDH values and confidence intervals [23]. These values are plotted as a heatmap using TBtools II v2.390 [24].

Orthologous gene cluster analysis was performed using the OrthoFinder [25]. Type Strain Genome Server (TYGS) was used to construct a phylogenomic tree for strain KR11^T^ and its closely related species. We retrieved the draft genome assemblies for closely related species from NCBI (https://www.ncbi.nlm.nih.gov/genbank/, accessed on 5 February 2026) and uploaded the user-submitted genomes on the TYGS (https://tygs.dsmz.de/user_requests/new, accessed on 5 February 2026). The results from TYGS include the phylogenomic tree by comparing the whole-genome assemblies, and a further tree was visualized using Interactive Tree of Life (iTOL) v7 (https://itol.embl.de/, accessed on 7 February 2026) [26].

## 3. Results and Discussion

### 3.1. Strain Characteristics and Physiology

**Figure 1:**
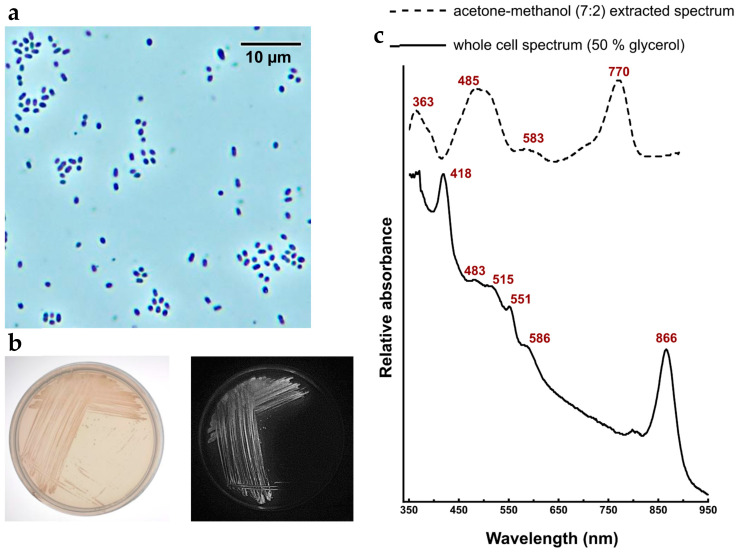
(**a**) Phase contrast image showing the rod-shaped cells (scale bar 10 μm). (**b**) Colonies of strain KR11^T^ showing beige colour (left), producing the BChl *a* autofluorescence (right) on agar plate. (**c**) Whole-cell absorption spectrum of strain KR11^T^ in 50% glycerol and absorption spectrum of acetone: methanol (7:2) extracted pigments.

Strain KR11^T^ cells are Gram-negative, motile, 1.0–2.2 μm long and 0.5–0.8 μm wide rods (Figure 1a). KR11^T^ colonies on agar plates are beige in colour and emit BChl *a* autofluorescence (Figure 1b). Liquid cultures of strain KR11^T^ exhibited photoorganoheterotrophic growth under both aerobic and microaerobic conditions (10% O_2_/90% N_2_) with a 12 h light:12 h dark cycle, utilizing organic carbon sources such as sodium pyruvate, malate, glucose, glycerol, and acetate. It also showed chemoorganoheterotrophic growth in the dark, with different organic carbon sources (Table 1). A weak anaerobic photoheterotrophic growth was observed after ten days of incubation, but photoautotrophic growth was not registered. Chemolithotrophy, photolithoautotrophy, and photolithoheterotrophy were not observed with sulfide or thiosulfate as electron donors. The colour of phototrophically grown cell suspensions was beige with a pink tinge due to the presence of pigments. Optimal growth occurs at 22–25 °C (range 20 to 40 °C) and pH 7.0 (pH range 6.0 to 9.0) under aerobic conditions, with a 12 h light:12 h dark light cycle. NaCl was not required for growth, but was tolerated up to 1.5% (*w*/*v*, NaCl). The strain KR11^T^ is positive for both oxidase and catalase activities.

Phenotypically, strain KR11^T^ can be distinguished from its closest validly published relative, *S. tardus* CYK-10^T^, by a combination of at least eight phenotypic and physiological traits (Table 1). Notably, KR11^T^ is motile and positive for catalase, whereas *S. tardus* CYK-10^T^ is non-motile and negative for catalase. In terms of carbon source utilization, KR11^T^ is able to utilize sucrose, trehalose, and rhamnose, none of which support growth in *S. tardus* CYK-10^T^. These differences in enzymatic activities and carbon utilization profiles collectively provide robust phenotypic evidence for the species-level distinctiveness of strain KR11^T^.

Strain KR11^T^ can also be clearly differentiated from *T. fusiformis* SY72^T^ by its phototrophic capacity—evidenced by the presence of BChl *a* (Figure 1c)—its lower optimum growth temperature, motility, and positive urease activity. Differentiation from *R. sediminis* N1^T^ is KR11^T^ supported by the presence of BChl *a*, positive for catalase and urease activities, and the inability to utilize galactose while in *R. sediminis*—absence of BChl *a*, negative for catalase and urease activities, and ability to utilize galactose as a carbon source. The optimum growth temperature for KR11^T^ is 22–25 °C, while the optimum growth temperature for *R. sediminis* is 27–35 °C.

### 3.2. Pigment and Lipid Composition

The in vivo absorption spectrum of KR11^T^ cells suspended in 50% glycerol shows maxima at 418, 483, 515, 586, and 866 nm (Figure 1c). The absorption band at 418 nm likely originates from the cytochrome *c*. The weak absorption maxima at 483, 515, and 586 nm document the presence of carotenoids. The 866 nm absorption band originates from BChl *a* present in the light-harvesting complex I. An absorption peak corresponding to the light-harvesting complex II was not observed. The absorption spectrum of acetone–methanol (7:2) extracted pigments (Figure 1c) gave maxima at 770 nm and 485 nm, indicating the presence of the BChl *a* and carotenoids of spheroidenone series, respectively.

The main respiratory quinone in KR11^T^ is Q-10. The polar lipids of strain KR11^T^ comprises phosphatidylethanolamine, phosphatidylglycerol, uncharacterized aminophospholipids, and uncharacterized phospholipids (Figure S1). The primary cellular fatty acids in strain KR11^T^ (>10%) were C_15:0_ anteiso (11.48%) and C_18:1_ ω7c (13.09%). The fatty acid profile of strain KR11^T^ differs sharply from its closest relatives (Table 2). In *S. tardus* CYK-10^T^, *T. fusiformis* SY72^T^, and *R. sediminis* N1^T^, C18:1 ω7c dominates at over 60% of total fatty acids—typical for *Paracoccaceae* [4,29]. In KR11^T^, it drops to 13.09%, co-dominated by C15:0 anteiso (11.48%), a branched-chain fatty acid absent from all three reference strains [30]. Temperature and medium can shift fatty acid proportions, but they do not generate major fatty acids outside a strain’s metabolic pathway [30,31]. The complete absence of C15:0 anteiso from those three relatives reflects a real biochemical difference. The KR11^T^ strain was grown under standardized conditions (R2A, 25 °C, mid-exponential phase) while comparative data for the reference strains were taken from the literature (Table 2). Branched-chain anteiso fatty acids are rare in *Alphaproteobacteria* and carry taxonomic weight at genus and species levels [31]. They also increase membrane fluidity at lower temperatures [30,31], since KR11^T^ grows optimally at 22–25 °C, lower than *T. fusiformis* (30–37 °C) or *R. sediminis* (27–35 °C), and was isolated from a temperate freshwater pond. The fatty acid profile provides chemotaxonomic support for treating KR11^T^ as a novel *Stagnihabitans* species.

### 3.3. 16S rRNA Gene and Phylogenomic Analysis

**Figure 2:**
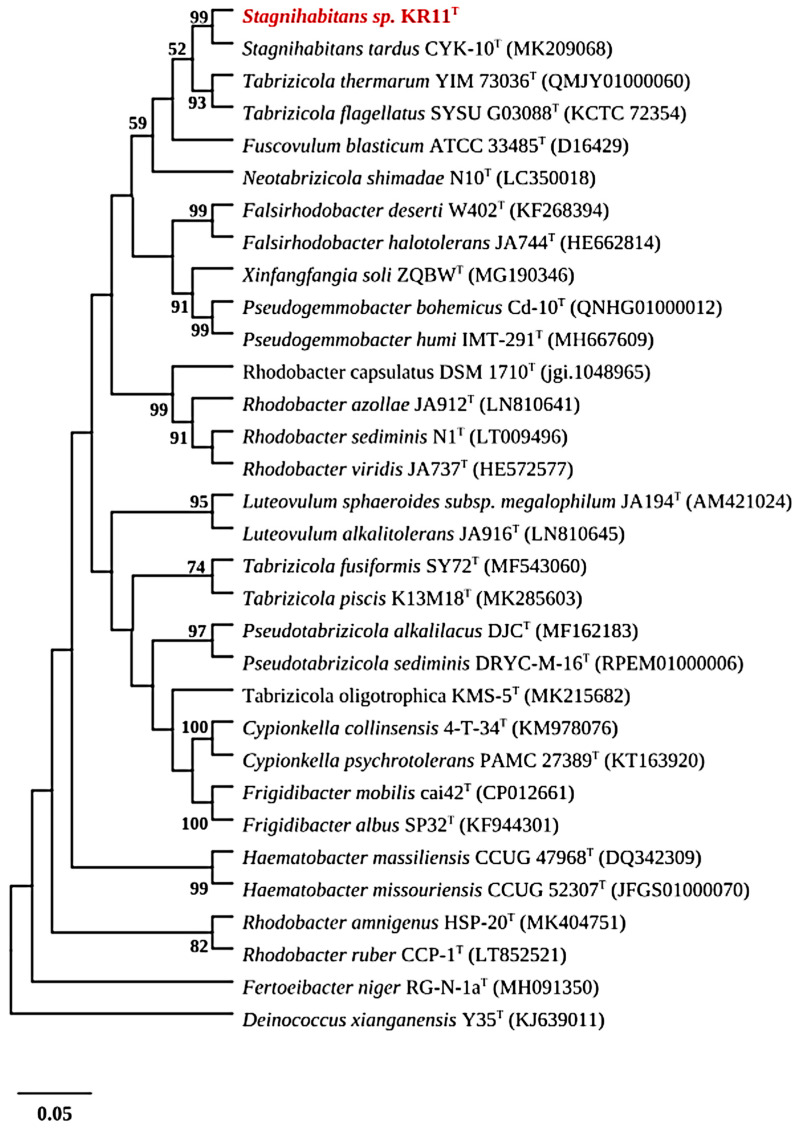
Phylogenetic relationship between strain KR11^T^ and closely related taxa within the family *Paracoccaceae*. The phylogenetic tree based on 16S rRNA gene sequence showing the relationship between strain KR11^T^ (in red) and closely related species was constructed by using the maximum likelihood (ML) method. Bootstrap values (>50%) based on 1000 resampling are shown. Bar, 0.05 substitutions per nucleotide position. *Deinococcus xianganensis* Y35^T^ was used as an outgroup.

The 16S rRNA gene of strain KR11^T^ (GenBank accession number MT586031) exhibits the highest sequence similarity with the 16S rRNA gene of *S. tardus* CYK10^T^ (98.84%), followed by *Tabrizicola thermarum* YIM 73036^T^ (96.60%) and *Tabrizicola fusiformis* SY72^T^ (95.95%). Phylogenetic analysis based on ML and NJ trees further revealed that the novel strain KR11^T^ formed a monophyletic clade with the genus *Stagnihabitans* and clustered with *S. tardus* CYK10^T^ within the family *Paracoccaceae* (Figure 2 and Figure S2).

**Figure 3:**
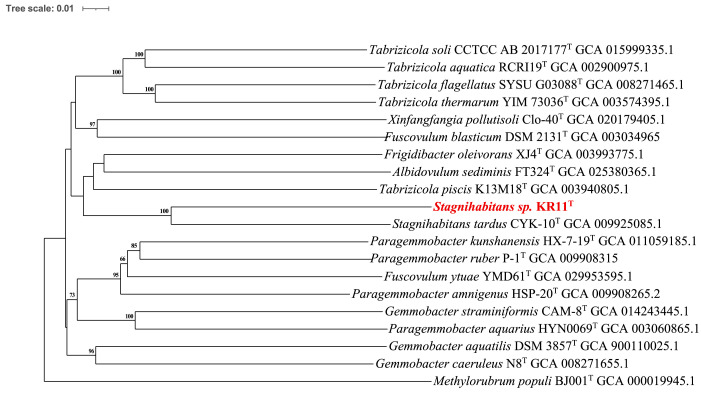
The phylogenomic tree was inferred using Genomic BLAST Distance Phylogeny (GBDP) implemented in the Type Strain Genome Server (TYGS). Branch lengths are scaled according to the GBDP distance formula d5, and bootstrap support values (>60%) based on 100 replications are shown at nodes. The tree was rooted using *Methylorubrum populi* BJ001^T^ as an outgroup. Strain KR11^T^ (in red) forms a distinct clade with its closest relatives. The tree was visualized and annotated using the Interactive Tree of Life (iTOL).

While the 16S rRNA gene sequence similarity between KR11^T^ and *S. tardus* CYK10^T^ (98.84%) does not follow the traditional thresholds of ~98.65% [32] and ~98.7% [33], such values are no longer considered definitive evidence of novel species. Chun et al. (2018) established minimal genomic standards for prokaryotic taxonomy, providing a framework under which 16S similarity alone is insufficient [33], particularly for genera such as *Nocardiopsis* [34,35,36] and *Streptomyces* [37,38], where closely related validly published species are known to share >99% 16S rRNA similarity and were resolved as distinct taxa via genomic metrics such as ANI and dDDH. Therefore, we employed phylogenomic analyses to clarify the status of KR11^T^. The phylogenomic tree was based on a total of 20 members of the family *Paracoccaceae*, which was rooted with one outgroup, *Methylorubrum populi* BJ001^T^. The resulting phylogenomic tree (Figure 3) and high bootstrap values confirmed the distinct lineage of the strain KR11^T^ within the *Stagnihabitans* genus and were clearly separated from *S. tardus* CYK10^T^, supporting its status as a novel species. The ANI values between KR11^T^ and its reference strains remained below 95% (the established species-delineation cutoff), further visualized in the heatmap provided in Figure S3. Furthermore, KR11^T^ exhibited dDDH values of 25.2% with *S. tardus* CYK10^T^, 19.2% with *T. flagellata* SYSU G03088^T^, 19.3% with *T. thermarum* YIM 73036^T^, and 19.0% with *F. blasticum* ATCC 33485^T^. dDDH values were found to be significantly below the 70% threshold [33,39], providing robust evidence that KR11^T^ is a distinct evolutionary lineage (Table S1 and Figure S4). The G+C difference in the draft genome of strain KR11^T^ with the closely related genome of its type strain showed up to 2% difference (Table S2). Along with the unique phenotypic traits, physiological profiles, and chemotaxonomic markers (fatty acids and polar lipids) collectively confirm the classification of KR11^T^ as representative of a novel species.

### 3.4. Genome Analysis

The draft genome of KR11^T^ consists of 21 contigs, with a total length of 4,085,976 bp. The quality of the draft genome was assessed using CheckM (v1.2.3), which reported 91.76% completeness and 3.8% contamination, meeting the minimum quality thresholds for a high-quality draft genome as defined by the MIMAG standards [40]. It contains one copy of each rRNA operon (5S, 16S, 23S), 50 tRNA genes, and 3790 protein-coding sequences. The G+C content is 65 mol%.

In addition, we calculated the number of shared orthologous gene clusters among strain KR11^T^ and the two reference strains *S. tardus* CYK-10^T^ and *T. fusiformis* SY72^T^. From the total number of orthogroups (3436), 69.2% (2379) were present in all three strains. Strain KR11^T^ shares 2903 and 2482 orthologous genes clusters with *S. tardus* CYK-10^T^ and *T. fusiformis* SY72^T^, respectively. The number of unique orthologous gene clusters (524) shared by strain KR11^T^ and *S. tardus* CYK-10^T^ was much greater than the number shared by either strain KR11^T^ and *T. fusiformis* SY72^T^ (103) or *S. tardus* CYK-10^T^ and *T. fusiformis* SY72^T^ (252), indicating a close relationship between strain KR11^T^ and *S. tardus* CYK-10^T^ at the genomic level. Therefore, phylogenetic and genomic analyses confirm that strain KR11^T^ is a distinct species within the genus *Stagnihabitans*.

The subsystems are annotated and classified based on their features and the number of counts provided (Figure S5). The maximum number of genes is assigned to protein and carbohydrate metabolism. A total of 35 genes have been assigned to virulence, disease, and defence subsystems, out of which 24 have been assigned to resistance to antibiotics and toxic compounds. A total of 15 genes were assigned to the metabolism of aromatic compounds, out of which nine were assigned to the metabolism of central aromatic compounds, and two were assigned to the peripheral pathway.

### 3.5. Metabolic Pathways

Similar to *S. tardus* CYK-10^T^, KR11^T^ contains a standard set of genes coding for enzymes of the tricarboxylic acid cycle (*gltA*, *acnA*, *icd*, *sucAB*, *sucCD*, *sdhABCD*, *fumC*, *mdh*) and genes coding for five main protein complexes involved in oxidative phosphorylation (marker genes *nuoABCDEFGHIJKLMN*, *sdhA*, *petABC*, *coxABC, atpIBEFHAGDC*), as well as all enzymes involved in the gluconeogenesis/glycolysis (marker genes *pckA*, *glpX*, *eno*, *tpiA*). Compared to the *S. tardus* CYK-10^T^, strain KR11^T^ lacks genes (*cydABX*) coding for cytochrome *bd* quinol oxidase (EC 7.1.1.7), a terminal oxidase in the respiratory chain. This enzyme is characterized by an extremely high oxygen affinity, playing a key role in adaptation to microaerobic environments [41].

It is notable that the KR11^T^ genome contains one continuous 47.3 kb photosynthesis gene cluster (PGC) involving 46 ORFs (locus tag: NX862_RS15560–NX862_RS15785) encoding genes for BChl biosynthesis, light-harvesting proteins, and the photosynthetic reaction centre subunits (genes *pufL* and *pufM*). An interesting feature is the presence of the oxygen-independent Mg-Protoporphyrin monomethylester cyclase (*bchE*) inside the PGC, while its oxygen-dependent form (*acsF*; EC 1.14.13.81) is at the periphery of the PGC. This arrangement is not common among phototrophic *Alphaproteobacteria* [42]. The PGC was found also in photoautotrophic *S. tardus* CYK-10^T^ (Table 3), but the main difference was in the ability to fix carbon dioxide. Strain KR11^T^ lacks genes for ribulose-1,5-bisphosphate carboxylase/oxygenase (RuBisCO) and other genes required for CO_2_ fixation. This is consistent with the fact that it cannot grow photoautotrophically. Therefore, strain KR11^T^ is obligately dependent on organic carbon sources and functions as a photoorganoheterotroph, potentially contributing to organic carbon cycling in its freshwater pond habitat rather than primary production. From nitrogen cycle pathways known from freshwater environments, both strains have the enzyme nitrous oxide reductase (*nosZ*; EC 1.7.2.4). It is a key enzyme in the denitrification pathway [43]. Due to the absence of other enzymes from the pathway, both organisms are rather N_2_O scavengers than full denitrifiers. Although the availability of phosphorus is usually not a limiting growth factor in freshwater eutrophic ponds, KR11 has a capacity to use non-conventional phosphorus sources using C-P lyase (*phnJ*; EC 4.7.1.1). Furthermore, we identified genes (*phnCDE*) coding for a transporter necessary for the transport of methylphosphonate into the cytoplasm, where it can by cleaved to methane and phosphate by the C-P lyase. Interestingly, the reference strain lacks genes for both the enzyme and the transporter. The main processes of the microbial sulfur cycle involve oxidation of reduced inorganic sulfur compounds and assimilatory and dissimilatory sulfate reduction. Both strains lack the sulfur-oxidizing (Sox) complex, which bacteria use for oxidation of thiosulfate, sulfite, or sulfide to sulfate to yield energy for growth [44,45]. Due to the absence of the gene *cysC*, coding for an adenylylsulfate kinase (EC 2.7.1.25), the assimilatory sulfate reduction pathway is incomplete in both strains. In the same token, the dissimilatory sulfate reduction pathway is also incomplete; only in the first step, the activation of sulfate to adenosine 5′-phosphosulfate is presumably functional in both organisms.

Strain KR11^T^ differs from *S. tardus* CYK-10^T^ also in several other genes, including those related to cell surface polysaccharide biosynthesis (*neuA*, *neuB*, *legB*), metal resistance (*arsC*), L-arabinose (*araFGH*), octopine/nopaline (*occPTOM*), cobalt (*cbiMNQO*), and copper (*copCD*) uptake, and the *attEFGH* ABC transporter. Strain KR11^T^ contains the complete flagellar gene cluster, which is in line with the observed motility. Strain KR11^T^ also contains CRISPR-associated proteins (*cas1*, *cas3*) and a type VI secretion system component (*tssM*). These genomic differences support that strain KR11^T^ is functionally distinct from *S. tardus* CYK-10^T^. Furthermore, KR11^T^ contains a complete, continuous gene cluster (locus tag: NX862_RS04510–NX862_RS04585) encoding gene transfer agents (GTAs), virus-like particles that transfer segments of genomic DNA between prokaryotic cells. The gene organization is similar to the GTA cluster in *Rhodobacter capsulatus* DSM 1710^T^ [26].

## 4. Taxonomic Conclusions

The strain KR11^T^ was distinguished from closely related strains, *S. tardus* CYK-10^T^, *T. fusiformis* SY72^T^, and *R.sediminis* N1^T^, by physiological, morphological, and biochemical characteristics such as cell size, cell shape, NaCl tolerance, enzyme activity, range of temperature for growth, acid production, and cellular fatty acid composition (Table 1). Based on combined phenotypic, chemotaxonomic, genomic, and phylogenomic evidence, strain KR11^T^ is proposed as a novel species within the genus *Stagnihabitans*.

### Description of Stagnihabitans lacustris sp. nov.

la.cus’tris. N.L. masc./fem. adj. *lacustris*, inhabiting a lake, referring to *Stagnihabitans lacustris* as a novel species predominantly found in lakes.

Cells are Gram-negative, motile, and rod-shaped. Colonies are small and beige-coloured on R2A agar plate after incubation for 24 h under aerobic conditions at 22–25 °C under a 12 h light:12 h dark cycle. A weak anaerobic photoheterotrophic growth was observed after ten days of incubation in contrast; substantial growth was observed in microaerophilic conditions (10% O_2_ + 90% N_2_) at 25 °C after three days. Optimum growth occurs at pH 7.0–7.2; NaCl is not required for growth but is tolerant to NaCl concentrations up to 1.5% (*w*/*v*). It can grow from 20 °C to 40 °C (optimum 22–25 °C). The in vivo absorption spectra of cells in 50% glycerol exhibited peaks at 418, 483, 515, 586, and 866 nm, documenting the existence of photosynthetic complexes. The absorption spectra of pigments extracted using acetone–methanol (7:2, *v*/*v*) displayed a peak at 770 and 487 nm, representing photosynthetic pigments BChl *a* and carotenoids of the spheroidenone series, respectively (Figure 1c). The main respiratory quinone is ubiquinone-10 (Q-10). The preferred growth mode is photoheterotrophy. Catalase and oxidase are present, but arginine dihydrolase, lysine decarboxylase, and arginine decarboxylase activities are absent. Gelatin, starch, chitin, casein, and Tween 80 are not hydrolysed. Polar lipids contain phosphatidylethanolamine; phosphatidylglycerol; uncharacterized aminophospholipids; and uncharacterized phospholipids (Figure S1). The major fatty acids (>10% of total) are C_15:0_ anteiso and C_18:1_ ω7c. It is negative for the reduction in nitrate to nitrite. It utilizes fructose, salicin, sucrose, rhamnose, trehalose, maltose, raffinose, mannose, arabinose, pyruvate, and does not utilize dextrose, adonitol, dulcitol, cellobiose, inulin, galactose, sorbitol, melibiose, and lactose.

The type strain KR11^T^ = CCUG 74777^T^ = LMG 31924^T^ was isolated from the eutrophic Kaprový pond in Třeboň, Czechia (48°59.47′ N, 14°46.86′ E). The DNA G+C content of the type strain is 65 mol%, consisting of 4,085,976 bp. The GenBank accession numbers for the 16S rRNA gene sequence and the draft genome sequence of strain *Stagnihabitans lacustris* sp. nov. KR11^T^ are MT586031 and JAPDGS000000000.1, respectively.

## Figures and Tables

**Table 1:** Differential properties between strain KR11^T^ and its closely related type strains of genus *Stagnihabitans*. The KR11^T^ data were taken from this study while the data for the rest of the strains were taken from the literature: *Stagnihabitans tardus* CYK-10^T^ [5,6], *Tabrizicola fusiformis* SY72^T^ [27], *Rhodobacter sediminis* N1^T^ [28]. All the reference strains mentioned contain Q-10 as the main respiratory quinone. +, positive; w, weakly positive; −, negative; nd, not determined.

Characteristics	KR11^T^	*Stagnihabitans* *tardus* CYK-10^T^	*Tabrizicola* *fusiformis* SY72^T^	*Rhodobacter* *sediminis* N1^T^
Colony colour	Beige	White	Translucent white	Reddish-brown
Cell shape	Rod-shaped	Ovoid to Rod-shaped	Fusiform-shaped	Rod-shaped
Cell size (µm)	0.5–0.8 × 1.0–2.2	0.6–0.8 × 1.4–2.2	0.2–0.5 × 1.3–2.1	0.5–0.7 × 2.1–4.0
Motility	+	−	−	+
Growth without NaCl	+	+	+	+
Optimum temperature for growth (°C)	25	25	30–37	27–35
Optimum pH for growth	7.0	7.0	6.0–7.0	6.5–8.0
DNA G+C content (mol%)	65	66	63.7	70.66
Bacteriochlorophyll *a*	+	+	−	−
Catalase	+	−	+	−
Oxidase	+	+	+	−
Urease	−	+	−	−
Methyl red	−	nd	−	+
Reduction in nitrate	−	nd	−	+
Citrate utilization	−	−	−	nd
Indole production	−	−	−	−
Utilization of				
Sucrose	+	−	+	+
Inulin	−	nd	nd	nd
Melibiose	−	nd	nd	−
Galactose	−	+	w	+
Fructose	+	+	w	+
Mannose	+	+	+	nd
Cellobiose	−	+	+	−
Trehalose	+	−	+	nd
Sorbitol	−	−	w	+
Rhamnose	+	−	+	−
Lactose	−	nd	nd	−

**Table 2:** Cellular fatty acid composition of strain KR11^T^ and closely related type strains. TR—traces.

Fatty Acid	KR11^T^	*Stagnihabitans tardus* CYK-10^T^	*Tabrizicola fusiformis* SY72^T^	*Rhodobacter sediminis* N1^T^
Hydroxy:				
C_10:0_ 3-OH	0.88	5.8	4.3	3.4
C_18:0_ 3-OH	2.41	4.4	4.5	1.4
Saturated:				
C_16:0_	2.07	8.0	2.2	4.9
C15:0 anteiso	11.48	-	-	-
C_18:0_	1.52	3.4	2.9	6.7
Unsaturated:				
C_18:1_*ω7c*	13.09	64.8	69.3	66.7
C_18:1_*ω7c*-11 methyl	-	5.9	13.9	1.6
C_18:1_*ω9c*	-	-	-	-
C_18:0_ 10-methyl	0.5	-	1.2	-
C_15:0_ iso 2-OH/C_16:1_*ω7c*	1.79	-	TR	-
C_19:1_*ω6c*/*ω7c*/C_19:0_ cyc *ω10c*	2.48	-	-	-

**Table 3:** Comparison of selected gene and metabolic pathways between strain KR11^T^ and *Stagnihabitans tardus* CYK-10^T^. +, present; −, absent.

Gene/Pathways	KR11^T^	*Stagnihabitans tardus* CYK-10^T^
Chlorophyll biosynthesis	+	+
Bacterial light-harvesting proteins	+	+
Photosystem II-type photosynthesis reaction centre	+	+
CMP-N-acetylneuraminate biosynthesis	−	+
Legionaminic acid biosynthesis	−	+
Lipopolysaccharide assembly	−	+
Multidrug resistance efflux pumps	+	+
Protein and nucleoprotein secretion system, type IV	−	+
Protein secretion system, type VI	+	+
AttEFGH ABC transport system	−	+
Copper uptake system CopCD	−	+
Tricarboxylate transport system	−	+
Bacterial chemotaxis	+	+
Global two-component regulator PrrBA in proteobacteria	+	+
Flavohaemoglobin	−	+
Carbon monoxide oxidation	−	+
CO_2_ fixation: Calvin–Benson cycle	−	+
Homogentisate pathway of aromatic compound degradation	−	−
Protocatechuate branch of beta-ketoadipate pathways	−	−
Central meta-cleavage pathway of aromatic compound degradation	−	−
CRISPR-associated protein Cas1	+	−
CRISPR-associated helicase Cas 3	+	−
Transposable elements (TniB, TniA, RepA, etc.)	−	+

## Data Availability

The GenBank/EMBL/DDBJ accession numbers for the 16S rRNA gene sequence and the whole-genome sequences of strain KR11^T^ are MT586031 and JAPDGS000000000.1 respectively.

## Supplementary Materials

The following supporting information can be downloaded at: https://www.mdpi.com/article/10.3390/microorganisms14051157/s1, Figure S1: Two-dimensional thin-layer chromatogram of the total polar lipids of strain KR11^T^. Figure S2: Phylogenetic relationship between strain KR11^T^ and closely related taxa within the family *Paracoccaceae*. Figure S3: Heatmap generated with orthoANI values calculated using OAT software. Figure S4: The dDDH values were calculated using GGDC and plotted using TBTools. Figure S5: The functional annotation is plotted as the number of counts assigned to the subsystem using RAST. Table S1: Pairwise dDDH values and percent G+C difference in strain KR11^T^ with selected type strain genomes. Table S2: Dataset of selected type strains to compare strain KR11^T^.

## Abbreviations

The following abbreviations are used in this manuscript:

ANI	Average nucleotide identity
BChl	Bacteriochlorophyll
dDDH	digital DNA-DNA hybridization
GGDC	Genome-to-Genome Distance Calculator
GTA	Gene transfer agents
iTOL	Interactive Tree of Life
RAST	Rapid Annotation using Subsystem technology
R2A	Reasoner’s 2A medium
TYGS	Type Strain Genome Server
QUAST	Quality Assessment Tool
Q-10	ubiquinone-10
