# Supplementary material for: Stagnihabitans lacustris sp. nov., an Anoxygenic Photoheterotrophic Bacterium of the Family Paracoccaceae, Isolated from a Eutrophic Pond in Czechia
Source: Microorganisms. 2026 May 20;14(5):1157. doi: 10.3390/microorganisms14051157 (PMC13209277; doi:10.3390/microorganisms14051157)
Supplement: Supplementary file 1 [file microorganisms-14-01157-s001.zip › microorganisms-4264775-supplementary.pdf]

Stagnihabitans lacustris sp. nov., an Anoxygenic Photoheterotrophic Bacterium of the Family *Paracoccaceae*, Isolated from a Eutrophic Pond in Czechia

Aditi Singh [1], Sumeeta Kumari [2], Gunjan Vasudeva [2], Mohit Kumar Saini [3], Anil Kumar Pinnaka [2], Karel Kopejtka [3], Michal Koblížek [3,*] and Nupur [1,3,*]

[1] Department of Life Sciences, Sharda School of Bioscience and Technology, Sharda University, Greater Noida 201306, India; 2023372186.aditi@dr.sharda.ac.in

[2] Microbial Type Culture Collection and Gene Bank (MTCC), CSIR Institute of Microbial Technology (CSIR-IMTECH), Chandigarh 160036, India; sumeetakumari20@gmail.com (S.K.); gunjanvasudeva22@gmail.com (G.V.); apinnaka@imtech.res.in (A.K.P.)

[3] Laboratory of Anoxygenic Phototrophs, Centre Algatech, Institute of Microbiology CAS, 37901 Třeboň, Czech Republic; saini@alga.cz (M.K.S.); kopejk00@alga.cz (K.K.)

* Correspondence: koblizek@alga.cz (M.K.); nupur.1@sharda.ac.in (N.)

Supplementary Materials

Figure S1. Two-dimensional thin-layer chromatogram of the total polar lipids of strain KR11^{T}. The (A) total lipids, (B) phospholipids, (C) glycophospholipids, and (D) aminolipids. 10% ethanolic phosphomolybdic acid for total lipid analysis, alpha-naphthol with ninhydrin agent for amino lipids, and molybdenum blue for phospholipids. Abbreviations: PE, phosphatidylethanolamine; PG, phosphatidylglycerol; APL1, uncharacterized aminophospholipids; PL1-PL6, uncharacterized phospholipids.

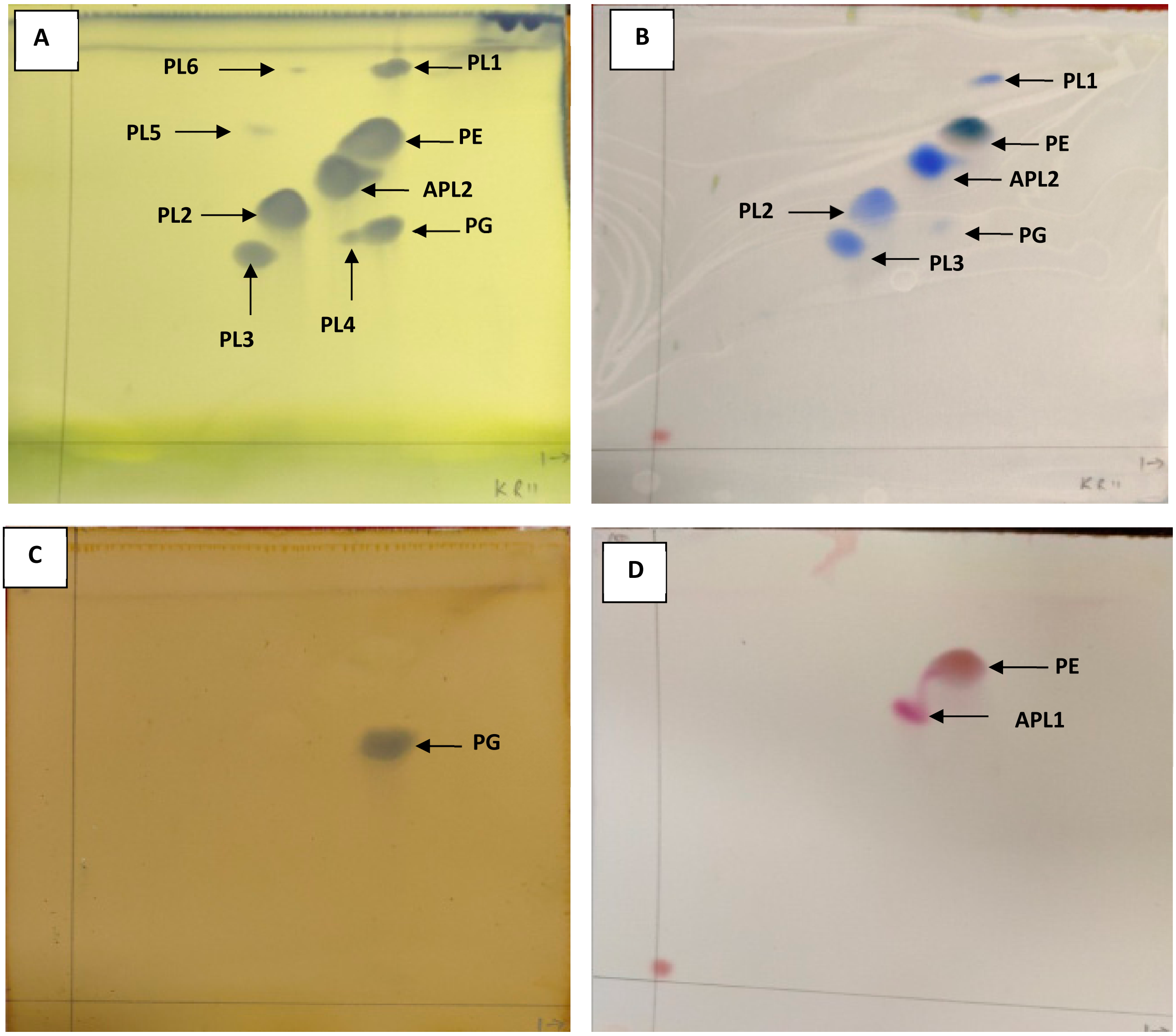

Figure S2. Phylogenetic relationship between strain KR11^T and closely related taxa within the family *Paracoccaceae*. The phylogenetic tree based on 16S rRNA gene sequence showing relationship between strain KR11^T and closely-related species was constructed by using the neighbour joining method. Bootstrap value (>50%) based on 1000 resampling are shown. *Deinococcus xianganensis* Y35^T was used as an outgroup.

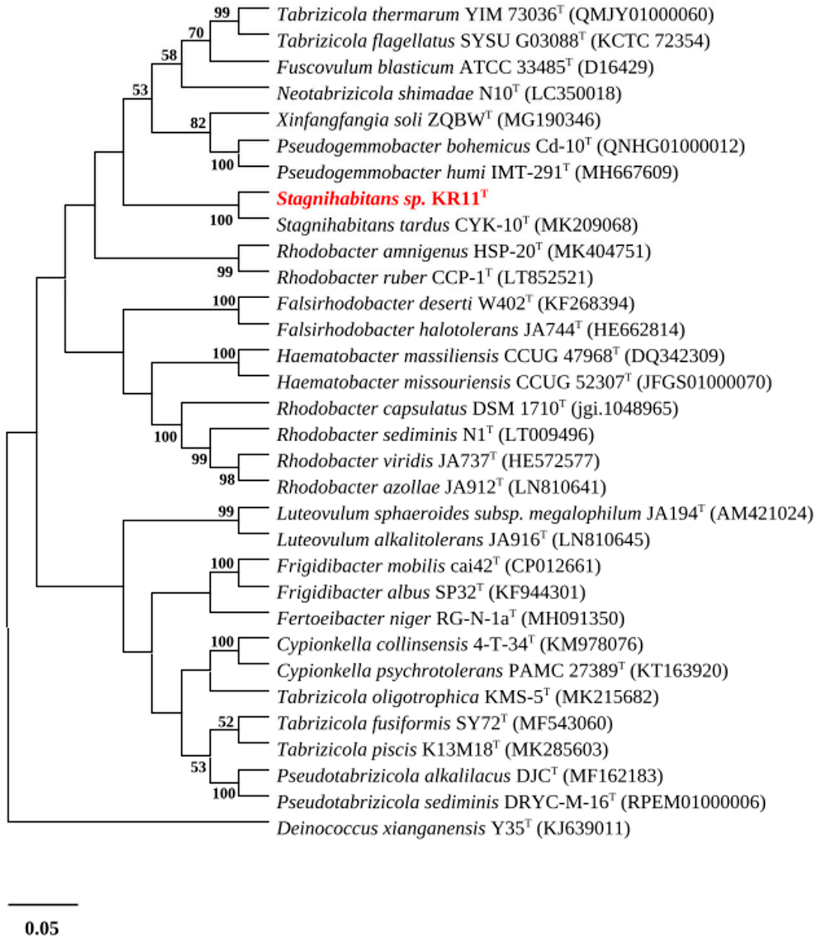

Figure S3. Heatmap generated with orthoANI values calculated using OAT software. The dataset contained the genomes of 10 type strains of related species. The genomes of closely related species were retrieved from EzBioCloud.net.

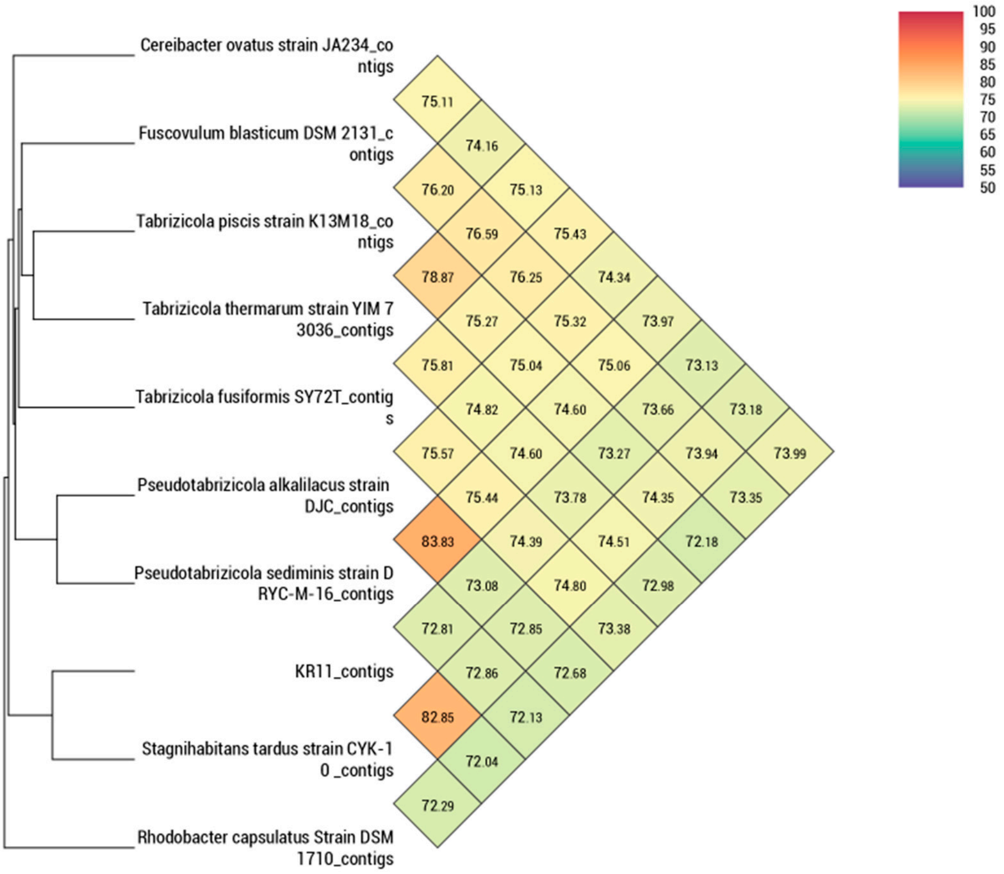

Figure S4. The dDDH values were calculated using GGDC and plotted using TBTools.

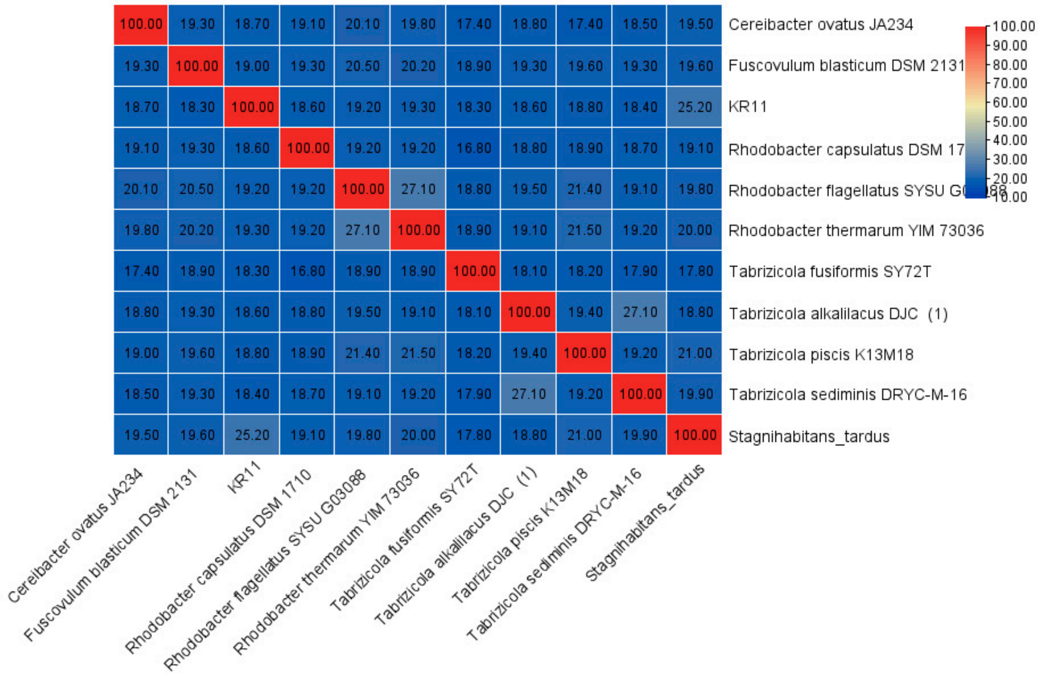

Figure S5. The functional annotation is plotted as the number of counts assigned to the subsystem using RAST.

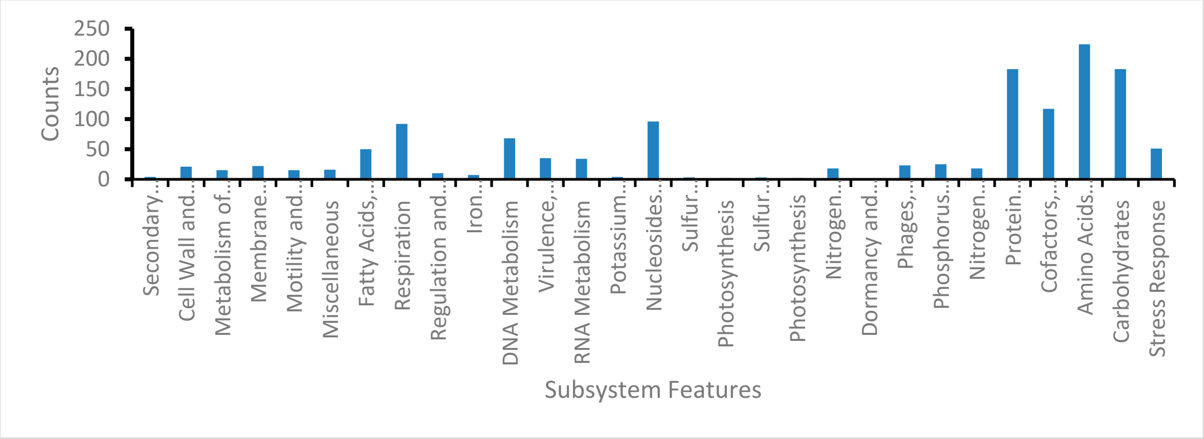

Table S1. Pairwise dDDH values and percent G+C difference in strain KR11^T with selected type strain genomes. The dDDH values are provided with the formula d4, the sum of all identities found in HSPs divided by overall HSP length.

Reference genome	d4 DDH (%)	G+C difference
Tabrizicola thermarum YIM 73036^T	19.3	1.72
Rhodobacter flagellatus SYSU G03088^T	19.2	2.63
Tabrizicola piscis K13M18^T	18.8	1.0
Rhodobacter capsulatus DSM 1710^T	18.6	1.57
Pseudotabrizicola alkalilacus DJC^T	21.1	1.96
Tabrizicola fusiformis SY72^T	18.6	1.91
Pseudotabrizicola sediminis DRYC-M-16^T	18.4	1.98
Cereibacter ovatus JA234^T	18.7	1.56
Stagnihabitans tardus CKY-10^T	25.2	0.96
Fuscovulum blasticum DSM 2131^T	19	1.56

Table S2. Dataset of selected type strains to compare strain KR11^T.

S. No.	Reference genome	Base pairs	Percent G+C	Number of proteins	Assembly accession no.
1.	*Tabrizicola thermarum* YIM 73036^T	3653867	66.79	3632	GCA_003574395.1
2.	*Tabrizicola piscis* K13M18^T	4387422	64.08	4263	GCA_003940805.1
3.	*Rhodobacter capsulatus* DSM 1710^T	3668822	66.54	3474	GCA_003254295.1
4.	*Pseudotabrizicola alkalilacus* DJC^T	4610061	63	4436	GCA_003443995.1
5.	*Tabrizicola fusiformis* SY72^T	528505	65.59	500	GCF_013328815.1
6.	*Pseudotabrizicola sediminis* DRYC-M-16^T	4040697	62.99	3899	GCA_004745575.1
7.	*Cereibacter ovatus* JA234^T	3806065	66.53	3617	GCA_900207575.1
8.	*Fuscovulum blasticum* DSM 2131^T	3588034	66.53	3531	GCA_003034965.1
9.	*Stagnihabitans tardus* CYK-10^T	4590930	66	4240	GCA_009925085.1
10.	Strain KR11^T	4085976	65	3817	GCF_025950525.1
